# Promoting the understanding of sex differences to enhance equity and excellence in biomedical science

**DOI:** 10.1186/2042-6410-1-1

**Published:** 2010-11-04

**Authors:** Arthur P Arnold

**Affiliations:** 1 Department of Integrative Biology and Physiology, University of California, Los Angeles

## 

From the moment of our conception, each of us has a sex. Sex has a major role in determining the physical attributes of our bodies, the structure of our brains, our behavioral tendencies, our susceptibility and reaction to diseases, the environment in which we grow up, our place in society, the attitudes of others towards us, and our conception of self. Although sex may be considered to be determined primarily biologically, our gender (i.e., the social perception and implications of our sex) is arguably equally or more important for our lives. Sex and gender differences are created by an intricate reciprocal interaction of numerous biological and environmental forces.

*Biology of Sex Differences *publishes articles concerning the role of sex and gender in normal traits and disease. As the official journal of the Organization for the Study of Sex Differences (http://www.ossdweb.org), *Biology of Sex Differences *will serve as a forum for discussion of the forces that make females and males different, and the downstream pathways that are affected by sex-specific forces. *Biology of Sex Differences *will publish articles on animal models and humans. The launch of *Biology of Sex Differences *is the result of several major developments in biomedicine in recent years.

## Sex matters

There is increasing evidence that males and females differ in their basic physiology, and in the susceptibility to and progression of diseases. The 2001 US Institute of Medicine report [1] concluded that sex matters in major diseases and response to therapy, and sometimes the effect of sex is profound. Women suffer from autoimmune diseases much more frequently than men. For example, women have a nine-fold greater incidence of systemic lupus erythematosus than men, and up to three times greater incidence of multiple sclerosis [2,3]. Males suffer more from some brain diseases such as attention deficit hyperactivity disorder, autism spectrum disorders, Tourette's syndrome, Parkinson's disease, etc. [4,5]. Sex differences occur in metabolic disease, hypertension, cardiovascular disease, cancer, and response to drugs [6-10]. In some cases, the sex differences are complex, changing over the lifespan or as a function of environment, suggesting that there are multiple sex-biased or sex-specific factors that need to be understood. At the very least, explaining the sex-specific causal variables should help physicians and patients anticipate important changes over the lifespan. More importantly, understanding these factors could uncover sex-specific protective forces that are attractive targets for novel therapies.

## Biomedical science has shown a strong sex bias and blindness to sex

Analysis of the scientific literature in numerous disciplines leads to the general conclusion that males are studied much more than females [12], in both animal and human research, so that the basic biology of medical texts may better reflect the biology of males than females. Drug development is based on research on males, even for diseases that are more prominent in females, and despite evidence that drug metabolism and efficacy differ in the two sexes [10,11]. Moreover, the sex of the experimental subjects is often not recorded in scientific reports [12]. For this reason, sex differences in physiology and disease are unappreciated simply because we don't have the data to know when differences occur, how large they are, and what causes them. Importantly, however, some sex-specific factors counteract each other [13], and make the two sexes more equivalent in phenotype rather than causing differences. For example, inactivation of one of the two X chromosome in every non-germline cell makes XX female cells more similar to XY male cells, in gene expression and function [14,15]. Thus, appreciating the impact of sex on cell function requires not just studying functions that are overtly different in the two sexes, but also those that are equivalent because of sex-specific factors that cancel each other out.

## The study of sex differences is a field of its own

Historically, sex differences have been studied most often in tissues involved in reproduction, where the differences are large: the gonads, internal and external genitalia, and brain and behavior. In the last century, these studies have given rise to general theories of sexual differentiation, about the genetic origins of sex differences, and the sex-specific signals that drive sexual differentiation of many tissues [16]. These studies result in a set of attitudes, animal models, and experimental protocols that can be applied to the study of sex differences of any tissue [17,18]. This common framework for studying sex differences is continuously evolving as it is broadened and applied to different research problems. *Biology of Sex Differences *will serve as a platform for ideas contributing to the evolution of the discipline.

Sex differences in physiology and disease will be front and center in *Biology of Sex Differences*, not a sub-topic. Unlike journals that focus on specific physiological systems or diseases, *Biology of Sex Differences *will aim to compare sex-specific effects across tissues, species, and diseases. The goal is to build on knowledge of sex differences in various systems to understand more fully how sex differences impact the entire organism.

## New understanding of the biology of sex differences

The revolution in molecular biology and genetics is rapidly advancing our understanding of the sex chromosomes, which are the only factors (in animals with heteromorphic sex chromosomes) that are thought to differ consistently in female and male zygotes, and which therefore give rise to all subsequent sex differences [16,19]. Understanding sex differences in phenotype and disease must therefore be related eventually to some fundamental differences in these chromosomes, which cause a constitutive sexual inequality in the expression of X or Y genes. Multiple X and Y genes cause sex differences, but most of them have not been identified [16,20]. Of primary importance in mammals is the Y chromosome gene *Sry*, which initiates molecular and cellular cascades in the undifferentiated gonads of males that lead to differentiation of testes [21]. In the female gonads, other pathways are activated that cause ovarian differentiation and suppress testicular development [22]. The sexual differentiation of the gonads leads to life-long differences in the levels of gonadal hormones, which cause many or most sex differences in other tissues [16,23]. The downstream pathways responding to sex-specific hormonal and genetic signals are complex, tissue- and age-specific, and poorly understood. *Biology of Sex Differences *will promote understanding of the sex-specific signals and their downstream pathways and effects, including their impact on disease processes.

## "Nothing in biology makes sense except in light of evolution"

Dobzhansky's [24] famous admonition holds true for the study of sex differences. The evolution of the mammalian genome only makes sense in light of the differences in the sex chromosomes, the differential fitness of alleles in the two sexes, and the co-evolution of molecular pathways in the two sexes. The evolution of the sex chromosomes, for example, is thought to set up contrasting selection pressures in the two sexes, so that understanding the organization of modern genomes requires an evolutionary perspective [25]. In mammals, some sex-specific forces in females can be understood as a response to the constraint of having two X chromosomes and ovaries, and other forces in males are likely to be an evolutionary response to having a single X, *Sry *and testes. Each sex is constrained into specific adaptations because of previously existing sex differences in the genome, physiology, and ecological niche. The study of evolution must be a central part of the biology of sex differences.

We can only appreciate sex differences in physiology and disease by a broad study of numerous animal models, because important principles are more obvious in some species than others. Not infrequently, study of non-mammalian model systems has revealed principles that were later found also to be true for mammals including humans, but were not as easily discovered in mammals [26].

## The importance of sex-specific environments

It is abundantly clear, from research and casual observation, that girls and boys are reared in fundamentally different environments, and schooled within a highly gendered framework of expectations. Because sex-specific environmental forces correlate so strongly with sex-specific biological factors, it is often impossible to separate their effects, especially in humans. Environmental factors causing sex differences are often routinely excluded or ignored by biologists because they have been considered to be outside of the realm of biology. Nevertheless, such forces interact strongly with biological factors. It is time to be inclusive, and purposely expand the biological investigation of sex differences to incorporate the interaction of environmental and biological factors. Especially now, as the epigenetic effects of environments on biological function are just beginning to be appreciated at the molecular level [27-29], the study of sex-specific environments must be incorporated into the biological study of sex differences.

## Studying sex differences is an important strategy for understanding diseases and for developing therapies

The existence of sex differences in disease implies that one sex has a sex-specific factor or process that protects from the disease. If that factor can be enhanced or modified, by direct manipulation of the factor or its downstream pathways, then the effects of the disease may be mitigated in both sexes. Because most factors causing sex differences are not lethal, they are attractive targets for drug development. The current lack of understanding of the biological basis of sex differences in disease may represent a large untapped reservoir of information relevant to development of novel therapies.

## Equality and Diversity in Clinical Practice

*Biology of Sex Differences *will serve as a forum for translating basic science into clinical practice. We welcome articles studying basic biology, that translate basic biology into medical practice, and that measure sex differences in the clinic and in the incidence and progression of human disease.

Sex and age are the two main modifiers of disease risk, presentation, and treatment. Treating one sex like the other may be as inappropriate as treating a child like an adult. To improve health for both women and men, we must understand specifically how sex modifies disease risk, presentation, and treatment. The equitable treatment of females and males requires an understanding of their differences, which provides a necessary foundation for an appropriate response to their individual differences. To ignore the differences is as dangerous as it is to overemphasize them. A goal of the *Biology of Sex Differences *is to increase understanding of the sex-specific forces that make the sexes different, and make them more similar than they otherwise would be.
